# The neurochemical pathology of schizophrenia: post-mortem studies from dopamine to parvalbumin

**DOI:** 10.1007/s00702-021-02453-6

**Published:** 2021-12-21

**Authors:** Gavin P. Reynolds

**Affiliations:** Biomolecular Sciences Research Centre, Sheffield Hallam University, Howard Street, Sheffield, S1 1WB UK

**Keywords:** Dopamine, Parvalbumin, Psychosis, GABA, Glutamate, DNA methylation

## Abstract

Research in Peter Riederer’s lab in Vienna in the late 1970’s came from a strong tradition in post-mortem neurochemical studies, at that time a relatively niche approach in neuroscience research. He was also early to recognise the value of post-mortem brain tissue in elucidating pharmacological mechanisms of neuropsychiatric treatments. I was fortunate to have Peter Riederer as a mentor in my early post-doctoral career; his generous support and the opportunities to use post-mortem brain tissue provided an invaluable grounding on which much of my future research was based. In this paper, I shall provide a brief overview of one trajectory of my research into the neurobiology of schizophrenia that started in the Riederer lab in Vienna investigating dopamine and the D2 receptor. Subsequent research to understand findings of increased dopamine resulted in the identification of reduced GABAergic innervation, culminating in the finding of a deficit in the parvalbumin-containing subtype of GABAergic neurons. Most recent work has been studying how changes in DNA methylation of the parvalbumin gene may relate to these findings in psychotic illness and its animal models.

## Introduction

There is no doubt that the study of the brain at post-mortem has been an invaluable approach to understanding brain disease. This is particularly true of neurodegenerative disorders; the current symptomatic treatment of both Parkinson’s (PD) and Alzheimer’s (AD) diseases owes everything to the findings of brain neurotransmitter deficits—dopamine in PD, leading directly to replacement therapy with levodopa, and acetylcholine in AD, indicating the potential for cholinesterase inhibition. In the late 1970s, I was at the beginning of my post-doctoral career and keen to apply post-mortem studies of the brain to my interest in understanding the neurochemical pathology of schizophrenia. To that end, I was particularly fortunate in having the opportunity to spend some time in Vienna with Peter Riederer as a mentor and research supervisor, working with him in the Ludwig Boltzmann Institute for Clinical Neurobiology at Lainz Hospital, where Birkmayer had led the clinical group identifying the dopamine deficit of PD and introducing levodopa therapy. This paper will describe some of my personal journey over more than 4 decades of research using post-mortem brain tissue, a journey which started with Peter Riederer in Vienna and reflects his strong influence on my approach to research.

## Dopamine D2-like receptors in schizophrenia

As well as allowing us to study directly the neurochemistry of disease, post-mortem brain tissue can also inform our understanding of mechanisms of drug action—the initial reason for my visit to the Riederer lab (Reynolds et al. 1978, 1980a). With this experience and the support of Peter and his colleagues I went on to study my primary interest—the brain in schizophrenia. At that time there was substantial effort aimed at investigating the role of dopamine receptors in the disease. There were two alternative views: that the apparent increase in dopamine D2 receptor density in the brain in schizophrenia was an aetiological factor, or that this increase was a consequence of treatment with the antipsychotic drugs which animal experiments showed to upregulate the receptors in brain tissue. We were able to test this using brain tissue taken at post-mortem from people with schizophrenia, finding an elevation in D2 receptors in patients who received antipsychotic drugs over those who did not (Reynolds et al. 1980b).

The dopamine receptor hypothesis did not go away so easily, however. The later identification of the D4 subtype of the “D2-like” dopamine receptors and their apparent elevation in the brain in schizophrenia reignited the discussion (Seeman et al. 1993). This finding, however, we were unable to replicate, showing the original observation was likely to be artifactual and again a consequence of antipsychotic drug treatment (Reynolds and Mason 1994).

## Dopamine and GABA

After moving to the MRC Brain Tissue Bank in Leslie Iversen’s Unit in Cambridge, I could draw on the valuable experience obtained in the Riederer lab in Vienna to develop post-mortem studies of brain disease. At that time there was some evidence suggesting that schizophrenic psychosis originated in the left temporal lobe (Flor-Henry 1969). Wanting to test this hypothesis in terms of neurotransmitter changes, I investigated limbic tissues in both brain hemispheres, finding that in schizophrenia the left amygdala had an elevation in dopamine (Reynolds 1983). It seemed unlikely that this reflected increased neuronal innervation, for which there was no evidence, rather than a regulatory effect on neurotransmitter activity. This argument led to the hypothesis that a deficit of inhibitory GABA neurons might underlie the increase in dopamine. We (Reynolds et al. 1990), and others (Simpson et al. 1989), found hippocampal deficits of a marker for GABAergic synapses, adding to the accumulating evidence that has now established the deficit or dysfunction of GABAergic interneurons as a key feature of the pathology of the brain in schizophrenia (Benes and Berretta 2001). In an intriguing finding supporting our hypothesis, these deficits appeared greater in the left hemisphere and correlated inversely with the changes in dopamine in the left amygdala (Reynolds et al. 1990), providing a possible mechanism relating neuropathology to a psychosis that responds to dopamine receptor antagonism.

## N-acetylaspartate and glutamate

These observations came at a time when there was growing evidence for a genuine neuropathology of schizophrenia, evidence pointing particularly to tissue loss in the neocortex and medial temporal lobe (e.g. Brown et al 1986). Such macroscopic abnormalities have their correlates in the evidence for neuronal deficits and dysfunction. A general indicator of neuronal activity and integrity that is not directly related to neurotransmitter function is N-acetylaspartate (NAA). The interest in NAA as a neuronal marker is in part because it can be detected in the living brain by magnetic resonance spectroscopy (MRS); analyses of NAA in brain tissue taken at post-mortem provide a useful correlate of such in vivo studies, albeit with far greater spatial resolution. Specific deficits in NAA have been identified in several brain regions (temporal cortex, hippocampus, amygdala) implicated in the pathophysiology of schizophrenia (Nudmamud et al. 2003; Reynolds and Reynolds 2011).

In addition to the findings implicating the GABAergic system, evidence was emerging for the involvement of another neuronal system in schizophrenia, that of glutamate. Interest in glutamatergic neurotransmission in this context developed from the “NMDA hypothesis” in which a hypofunction of the glutamate-NMDA receptor was thought to underlie the symptoms of schizophrenia (Olney et al. 1999). An early post-mortem study of NMDA receptors in schizophrenia undertaken in the Riederer lab identified increases in ligand binding to the NMDA receptor which reached significance in the striatum (Kornhuber et al. 1989). From the observation of deficits in pre-synaptic glutamatergic markers, we later interpreted this finding as an upregulation in response to a loss of cortico-striatal glutamatergic input (Nudmamud-Thanoi et al. 2007). While the original hypothesis derived from the validity of animal models involving NMDA antagonism in which correlates of positive, negative and cognitive symptoms could be observed, much further evidence has accumulated supporting the involvement of glutamate neurotransmission in schizophrenia. This ranges from genetic variation in several glutamate receptors associated with the disease (GRM3, GRIN2A, GRIA1) to MRS determination of glutamate and post-mortem findings of changes in pre-synaptic markers of glutamatergic synapses (reviewed by Uno and Coyle 2019).

As the major inhibitory and excitatory neurotransmitters in the human brain, glutamate and GABA are intimately interrelated and a change in one is likely to influence the activity of the other. Thus a dysfunction of glutamate may, as a consequence of the presence of NMDA receptors on some GABAergic neurons, affect their activity, while GABAergic interneurons are also involved in the inhibitory regulation of glutamatergic output.

## GABA and parvalbumin

Perhaps our most exciting findings relating to the neuronal pathology of schizophrenia came about from our attempts to better understand the GABAergic deficits. GABA-containing neurons are not all the same; they can be subdivided in several ways including on the basis of structure, of co-existing peptide transmitter or of calcium binding protein (CBP) present. Some previous post-mortem studies of neuropeptides localised in GABAergic neurons had suggested selective limbic deficits of these neurons in schizophrenia (Roberts et al. 1983). Of the three CBPs defining GABAergic neurons in the human brain, parvalbumin (PV) is expressed relatively late during development after the establishment of synaptic contacts (Solbach and Celio 1991), leading us to hypothesise that there might be an early 'window of vulnerability' during which these neurons may be sensitive to e.g. excitotoxic damage, resulting in a deficit in these GABAergic cells in people with schizophrenia. This is indeed what we found in the brain at post-mortem, reporting reductions of over 30% in frontal cortical PV-containing neurons (Beasley and Reynolds 1997). In studies of other regions, we observed even greater deficits of 60% in hippocampal tissues (Zhang and Reynolds 2002), certainly one of the most robust of the neurochemical changes in the brain in schizophrenia.

We went on to demonstrate that a neonatal inflammatory challenge, with the bacterial endotoxin lipopolysaccharide, could reduce hippocampal PV-immunoreactive cells in adult rats, confirming that developmental trauma could influence PV neuronal density (Jenkins et al. 2009). However we also demonstrated that a sub-chronic regime of phencyclidine (PCP) administration, modelling some cognitive aspects of schizophrenia, reduced PV expression in the adult rats (Abdul-Monim et al. 2007), indicating that a pharmacological model of schizophrenia could affect PV and questioning whether the effect in the disease was of developmental origin. Further animal models including post-weaning social isolation (Harte et al. 2007) and methamphetamine administration (Veerasakul et al. 2016) also demonstrated PV deficits, indicating a general association of these cellular changes with models of psychosis rather than solely an early-life vulnerability in schizophrenia. However, that this might provide a mechanism for dopaminergic dysfunction is indicated by other findings demonstrating that PV deficits can result in elevated dopaminergic activity (Boley et al. 2014).

## Parvalbumin epigenetics

Whether the PV-containing neurons were lost in schizophrenia or had only reduced their expression of PV protein was unclear. That there might be some reversibility in the PV deficit seen in some animal models of schizophrenia suggested the latter interpretation was true. To explore the control of PV expression and how it might be affected by various pharmacological or developmental challenges, we focused further effort on an epigenetic control mechanism: that of promoter sequence DNA methylation. DNA methylation is a dynamic process that can potentially bridge the gap between genetic and environmental factors in the pathology of schizophrenia; methylation of promoter sequences can influence transcriptional regulation by disrupting the binding of proteins, such as transcription factors or histones, to DNA. A previous study showed the neurotoxic effect of manganese increased methylation of the mouse PV gene and reduced the numbers of PV-immunoreactive neurons (Wang et al. 2013); we asked whether PV gene (PVALB) methylation was elevated models of schizophrenia as well as in human psychotic illness.

This is indeed what we observed; methylation of sites in a sequence of the PVALB promoter region in humans was elevated in the hippocampus in schizophrenia (Fachim et al. 2018). We could identify an equivalent methylation site in the rat genome; this was also hypermethylated in the PCP model (Fachim 2016). Furthermore PVALB methylation was increased in blood-derived DNA from methamphetamine users with a history of psychosis (Veerasakul et al. 2017). These findings provide strong evidence for a consistent relationship between increased methylation at a particular site on the gene coding for PV and PV deficits associated with psychotic illness and its models. It is tempting to assume that this is a causal effect: that pharmacological, genetic or environmental influences result in increased PVALB methylation which in turn is responsible for a reduction in PV expression and a resultant disruption of GABAergic interneuron function. However, the direction of causality is not yet clear and evidence for this relationship is so far only circumstantial. Nevertheless, the findings are intriguing, the more so when it is apparent that the methylation sites affected may disrupt the binding of important transcription factors involved in oxidative stress (Nrf2) (Fachim et al. 2016) and neuronal function (CREB) (Fachim et al. 2018).

## Conclusions

**Fig. 1:**
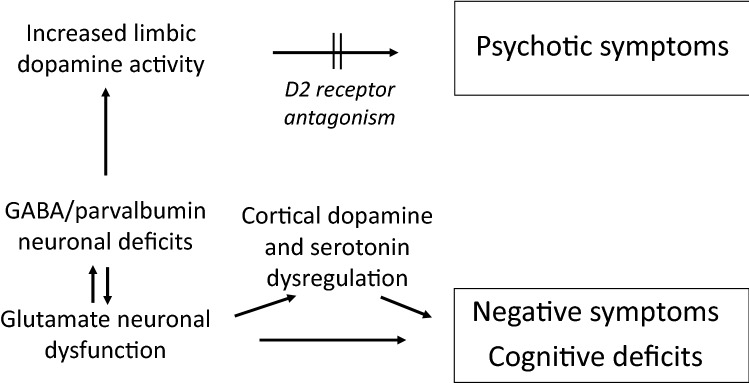
Neurotransmitter processes in schizophrenia

Although the advances in neurobiological research over the past 4 decades have been huge, there remains much to understand about brain dysfunction in schizophrenia. This paper has covered a very small part of this research, with an inevitably limited perspective. Nevertheless, the identification of highly specific changes in subgroups of neurons, notably including the parvalbumin/GABA interneurons that have an important role in the inhibitory control of glutamatergic output, provides some elucidation of the relationships between neurotransmitter dysfunction and symptoms in schizophrenia (Fig. 1) as well as identifying potential new targets for the treatment of psychotic illness.

Given the complexity of brain function, the interplay between glutamate and GABA is, inevitably, not the only involvement of neurotransmitter systems in the pathophysiology of schizophrenia. As indicated above, a consequent hyperactivity of limbic dopamine mediates the positive symptoms of schizophrenia. Other transmitter systems may also influence the cognitive and negative symptoms by mediating or modulating the effects of glutamate and GABA; although not reviewed here, there are strong indications of a frontal cortical dopaminergic hypofunction as well as the involvement of serotoninergic systems in these symptoms. As yet, and unlike the efficacy of dopamine D2 antagonism in relieving positive symptoms, these have not provided effective pharmacotherapeutic targets. Nevertheless, the findings reviewed here contribute to the conclusion that post-mortem brain tissue provides a research tool which has much to offer in our search for a clearer understanding of this devastating disease.
